# Prenatal Human Immunodeficiency Virus Testing and Patient Management by Obstetricians in a High Seroprevalence Community

**DOI:** 10.1155/S1064744994000359

**Published:** 1994

**Authors:** William R. Robinson, Michael Fleischer

**Affiliations:** Department of Obstetrics and Gynecology Box SL-11 Tulane University School of Medicine 1430 Tulane Avenue New Orleans LA 70112 USA

## Abstract

*Objective:* In order to determine the practice habits of obstetricians concerning frequency of prenatal human immunodeficiency virus (HIV) testing and management strategies for HIV-seropositive obstetric patients, we conducted a telephone survey of practicing obstetricians over a 3-month period.

*Methods:* In the New Orleans metropolitan area, 71/104 (68%) obstetricians participated and completed the survey.

*Results:* Of these obstetricians, 43/71 (60.6%) test all new obstetric patients for HIV; 64/71 (84.5%) routinely ask the patients about risk factors for infection; and 28/71 (39.4%) have actually cared for an HIV-positive patient in their practice. Those obstetricians who routinely tested for HIV were more likely to have personally managed an infected patient and more likely to ask about risk factors. The number of obstetricians who would manage infected patients without consultative assistance was 8/71 (11%).

*Conclusions:* We concluded that obstetricians in this community have largely accepted routinely offered prenatal testing and risk assessment, but they have assumed a relatively small role in risk reduction counseling and treatment.


Infectious Diseases in Obstetrics and Gynecology 2:25–29 (1994)
© 1994 Wiley-Liss, Inc.

### Prenatal Human Immunodeficiency Virus Testing and Patient Management by Obstetricians in a High Seroprevalence Community

**William R. Robinson and Michael Fleischer**

*Department of Obstetrics and Gynecology, Tulane University School of Medicine, New Orleans, LA*

### Abstract

***Objective:*** **In order to determine the practice habits of obstetricians concerning frequency of prenatal human immunodeficiency virus (HIV) testing and management strategies for HIV-seropositive obstetric patients, we conducted a telephone survey of practicing obstetricians over a 3-month period.**

***Methods:*** **In the New Orleans metropolitan area, 71/104 (68%) obstetricians participated and completed the survey.**

***Results:*** **Of these obstetricians, 43/71 (60.6%) test all new obstetric patients for HIV; 64/71 (84.5%) routinely ask the patients about risk factors for infection; and 28/71 (39.4%) have actually cared for an HIV-positive patient in their practice. Those obstetricians who routinely tested for HIV were more likely to have personally managed an infected patient and more likely to ask about risk factors. The number of obstetricians who would manage infected patients without consultative assistance was 8/71 (11%).**

***Conclusions:*** **We concluded that obstetricians in this community have largely accepted routinely offered prenatal testing and risk assessment, but they have assumed a relatively small role in risk reduction counseling and treatment.** © 1994 Wiley-Liss, Inc.

Key words

HIV testing, obstetric practices, risk counseling, referral patterns

The number of women with the diagnosis of acquired immunodeficiency syndrome (AIDS) or human immunodeficiency virus (HIV) seropositivity in the United States continues to rise. As of March 1993, 11.9% of all cases of AIDS were in women. For the year preceding March 1993, nearly 14% of all new diagnoses of AIDS were made in women, nearly 80% of whom were of childbearing age. Louisiana ranked 7th in the United States in case rate per 100,000 population in March 1992, with 62% of cases (2,101/3,362) found in the New Orleans metropolitan area.[1,2] The impact of this problem on obstetric care includes concerns about perinatal transmission, retroviral and other therapies, and health care personnel exposure as addressed recently by the American College of Obstetricians and Gynecologists (ACOG).[3]

The accelerating case rate of AIDS in women nationally as well as in Louisiana indicates that practicing obstetricians are increasingly likely to be called upon to deliver care to HIV-infected patients. Currently, no state or local statutes concerning HIV testing of women in pregnancy are in effect in this jurisdiction. In addition, the local and state medical societies have not taken positions on these issues. As a result, no clear standard appears to exist regarding HIV testing in pregnancy in this

Address correspondence/reprint requests to Dr. William R. Robinson, Department of Obstetrics and Gynecology, Box SL-11, Tulane University School of Medicine, 1430 Tulane Avenue, New Orleans, LA 70112.

Clinical Study

Received January 10, 1994
Accepted April 7, 1994

community. In order to assess community practice standards and physician attitudes concerning HIV infection, we took a survey of practicing obstetricians in the New Orleans metropolitan area. This report is a summary of practice habits, the demographics of surveyed physicians, and a sampling of attitudes toward obstetric care of these women as expressed by the clinicians involved.

### MATERIALS AND METHODS

Currently practicing obstetricians in the New Orleans metropolitan area were surveyed by telephone as part of a project initiated by the Tulane-Louisiana State University Pediatric AIDS Clinical Trials Unit (PACTU) to increase awareness in the community and determine referral patterns of practitioners. Sources used for compiling the survey group included physicians listed by the Greater New Orleans Obstetrics and Gynecologic Society, the Tulane and LSU obstetric/gynecologic residency alumni associations, and physicians advertising in the yellow pages under the heading of obstetrics and gynecology. All physicians identified as practicing obstetrics were included in the survey group. This determination was made at the initial contact by confirming with the physician's office staff that he or she maintained an office-based obstetric practice and provided intrapartum services to his/her patients in a hospital or birthing center setting. Physicians who saw only gynecologic patients or did not provide care during labor and delivery were excluded.

By these methods, 104 area obstetricians were identified as being eligible for the survey. This group was then contacted by telephone during regular office hours between April 1993 and June 30, 1993. All obstetricians contacted were informed at the outset that the interviewer was a physician associated with the PACTU and that information obtained during the interview would be used to assess community practice patterns only, with no unique identifiers included. Of the eligible obstetricians 68% (71/104) agreed to participate and completed the interview, a rate comparable to others reported previously.[4,5] The most commonly given reasons for refusal to participate were lack of time and unwillingness to be identified, even though potential participants were given assurances of confidentiality prior to beginning the interview.

All interviews were conducted following a predetermined format. Responses were recorded on standardized precoded forms at the time of the interview. The mean number of calls made to complete each interview was 3, and the mean interview time was 9 min.

The participating physicians were asked if they offered HIV testing to all obstetric patients, if they identified patients with risk factors for HIV infection, and, if so, whether they used direct questioning or a preprinted questionnaire to do so. The obstetricians were also asked if they would manage an HIV-infected obstetric patient personally or if they preferred to refer such patients to other physicians and why they would refer. In addition, the physicians who stated they would personally manage HIV-infected patients were asked if they would obtain consultative help from other physicians. All the physicians were also asked if they had personally managed the prenatal care and delivery of an HIV-infected patient in their practice. The demographics of the participants were also recorded, including the practice setting, (solo, group, academic, HMO), the racial distribution and payment sources of their patients, their delivery volume per month, and the length of time each had been in practice.

Statistical analysis of the survey results was done using the Mann-Whitney U, Fisher's exact, and chi-square tests.

### RESULTS

The demographics of the surveyed obstetricians are summarized in Table 1. Of note, approximately 90% were in private practice in either group or solo settings. Nearly two-thirds (64.6%) of the patients seen were white, and nearly 80% were privately insured. Of those surveyed, 39% reported that they had treated HIV-infected patients.

In Table 2, 60.6% (43/71) of the obstetricians surveyed reported that they offered testing to all new obstetric patients for HIV using standard enzyme-linked immunosorbent assay (ELISA) testing with confirmatory Western blot. The number who reported having an HIV-positive patient in their practice was 28/71 (39.4%). A total of 64/71 (84.5%) reported that they asked patients whether they had any of the following risk factors: personal history of intravenous (IV) drug use, multiple sex partners (>3), a bisexual or drug-using partner, or a personal history of blood transfusions. However,

TABLE 1. Characteristics of surveyed obstetricians (N = 71)

| | | |
|---|---|---|
| Male | | 55 (77.5%) |
| Female | | 16 (22.5%) |
| Time in practice | | 16.6 years (range 1–43) |
| Deliveries/month | | 12.8 (range 3–30) |
| Had an HIV-positive patient | | 28 (39%) |
| Practice characteristics | | |
| Group | 43 | (60.6%) |
| Solo | 21 | (29.6%) |
| Academic | 7 | ( 9.8%) |
| Patient race (mean) | | |
| White | | 64.6% |
| Black | | 31.6% |
| Hispanic | | 2.7% |
| Asian | | 1% |
| Payment source | | |
| Private insurance | | 79% |
| Medicaid | | 20% |
| Self-pay | | 1% |

TABLE 2. Practices concerning HIV among surveyed obstetricians (N = 71)

| | |
|---|---|
| Those who routinely test for HIV | 43 (60.6%) |
| Those who have had HIV-positive patients | 28 (39.4%) |
| Those who routinely ask about patient risk factors | 64 (84.5%) |
| By printed questionnaire | 60 |
| By personal interview | 4 |
| Management of HIV-positive patients | |
| Refer to another physician | 32 (45%) |
| Obtain consultation with specialists | 31 (43.7%) |
| Alone | 8 (11%) |
| Reasons for referral (N = 32) | |
| Lack of expertise | 20 (62.5%) |
| Detrimental to practice | 9 (28%) |
| Risk of health care personnel | 3 (9.3%) |

the majority (60/64) of those physicians who asked about risk factors reported that they did so in the form of a preprinted questionnaire filled out by the patient prior to seeing the doctor. Very few (4/64) stated that they personally asked patients about specific risk factors.

When asked how they would manage an asymptomatic HIV-positive obstetric patient, 45% (32/71) stated they would refer the patient to another physicians for care; 43.7% (31/71) said they would care for the patient themselves with the aid of consultants, including perinatologists and infectious disease specialists; and 11% (8/71) said they would manage such patients alone. Three specific reasons were expressed by obstetricians who stated that they would refer HIV-positive patients elsewhere. The most frequent reason for referral was a lack of knowledge or expertise on the part of the obstetrician concerning HIV infections (20/32, 62.5%). The number who felt that treating HIV-positive patients would be detrimental to their practice because other patients would be reluctant to receive care from the same doctor was 9/32 (28%). The number who expressed concern over exposing health care personnel to HIV-infected patients was 3/32 (9.3%).

TABLE 3. Characteristics of obstetricians who test for HIV vs. non-testers[a]

| | Routine testing | |
|---|---|---|
| | Yes | No |
| Cared for HIV-positive patient | 56% | 6% (*P* = 0.0005) (95% CI 2.2–25.6) |
| Asked about risk factors | 93% | 71% (*P* = 0.019) (95% CI 1.2–22.3) |
| Group practice | 53% | 71% (NS) |
| Years in practice (mean) | 15.2 (SE = 1.5) | 16.8 (SE = 2) (NS) |
| Deliveries/month | 13.6 (SE = 1.0) | 11.9 (SE = 0.9) (NS) |
| Would refer HIV patients | 60% | 54% (NS) |

[a]CI = confidence interval; SE = standard error; NS = non-significant.

Table 3 summarizes the characteristics of those obstetricians who test for HIV vs. those who do not. Obstetricians who offer testing for HIV were significantly more likely to have actually cared for an HIV-positive patient (56% vs. 6%) [$P$ = 0.0005, 95% confidence interval (CI) 2.2–25.6]. In addition, those who tested were significantly more likely to ask patients about possible risk factors (93% vs. 71%) ($P$ = 0.0199, 95% CI 1.2–22.3). The 2 groups were similar in all other characteristics surveyed. The patient populations managed by the 2 groups were also similar in terms of racial makeup and reimbursement sources.

### DISCUSSION

Several attempts to document physician attitudes and practices regarding HIV-infected patients have been reported from a variety of locations in the United States during the AIDS era.[4,6–10] Most have demonstrated that a significant number of physicians in this country express a lack of knowledge or understanding of HIV infections. The percentage of physicians stating that they refuse to treat HIV-positive patients has ranged from 16% of family physicians in Oregon[7] to 23% of primary care physicians in Arkansas.[9] The current report has attempted to document the attitudes and practices of obstetricians providing care in a relatively high seroprevalence community.

We also found that physicians who routinely offered testing for HIV to obstetric patients were much more likely to state that they had personally treated HIV-positive patients (56% vs. 6%). Of interest is the fact that the individual and practice demographics of those who tested routinely were similar to those who did not, including experience, sex, practice volume, patient racial distribution, and reimbursement patterns. This would suggest that the patients in these practices should represent the same community and have similar rates of HIV infection. An explanation for the apparent dissimilarity could be that those physicians who are not testing routinely are simply not identifying those patients who are HIV positive, and the rate of infection is underreported. An alternative explanation would be that the dissimilarity is a result of predetermined selection bias. It is possible that physicians who had encountered an HIV-positive obstetric patient in their practice subsequently began testing all patients as a result. The motivation for testing was not specifically addressed during the interview.

Obstetricians who tested for HIV were also more likely to ask patients about possible risk factors for infection, although a relatively small number of the testing group (4/40) and none of the non-testing group (0/24) who determined risk factors used personal interviews to do so. The majority (60/64) used preprinted forms filled out by the patient. The effectiveness of a questionnaire compared with a personal interview for eliciting HIV-related behavior has not been clearly addressed. It has been documented that, when testing is performed only on patients who identify themselves as belonging to a risk group, significant numbers of infected women are not identified.[11] The likelihood that a personal interview will more accurately identify patients at risk for infection than will a printed questionnaire is unknown.

The rate of obstetricians who stated they would refer patients elsewhere (45%) was somewhat higher than the rate reported for all Louisiana primary care physicians (30%).[6] The reasons given for referral by obstetricians were similar to those given by primary care physicians. A lack of knowledge or expertise regarding HIV infection was expressed most commonly as a reason for referral (62.5%), followed by a fear of the practice being hurt by treating HIV-positive patients (28%). Risk of exposure to health care personnel was cited less frequently by the obstetricians than primary care physicians (9.3% vs. 20%).

These data suggest that the majority of obstetricians in this community use medical considerations as the primary basis for making treatment decisions concerning HIV-positive patients. More than 80% of those surveyed stated that they would care for such patients themselves, with or without consultation, or they would refer the patient to another physician because of their own lack of expertise. A minority would refer patients solely based on fear of exposure or stigmatization of the practice. These findings are consisted with occasional incidents of discrimination reported in obstetric-gynecologic offices involving HIV-positive women.[12–14]

These findings also show that approximately 85% of obstetricians surveyed routinely ask patients about risk factors for HIV infection, which is in excess of the 75% goal for the year 2000 set by the U.S. Department of Health and Human Services (DHHS).[15] However, as the majority of those surveyed use questionnaires rather than personal interviews for this purpose, it remains unclear how frequently obstetricians engage in counseling regarding risk reduction, which is also a component of the DHHS goal. In contrast, only 11% of those surveyed indicated that they would manage an HIV-positive patient without consultative help from specialty trained physicians. This may suggest that even those obstetricians who would not refer the patient frequently do not feel comfortable with their own level of knowledge and may be allowing other physicians to assume the major role in making treatment decisions.

In summary, it appears that obstetricians in this community have largely accepted routinely offered prenatal screening for HIV as well as commonly practiced infection risk assessment. However, the same findings suggest that obstetricians have not played a major role in risk reduction counseling or treatment. In order to better function as primary care providers to women, a recently stated goal of the ACOG,[16] we need a more complete assessment of the role of the specialist in caring for patients at risk for infection with HIV.

### REFERENCES

1. U.S. Department of Health and Human Services: HIV/AIDS Surveillance Report. Washington, DC: U.S. Government Printing Office, May 1993.
2. Delta Region AIDS Education and Training Center: Louisiana Surveillance Report, June 1993.
3. Human immunodeficiency virus infections. ACOG Tech Bull 169, June 1992.
4. Boekeloo BO, Rubin DC, Coughlin SS, Labbok MH, Johnson JC: Knowledge, attitudes and practices of obstetricians-gynecologists regarding the prevention of human immunodeficiency virus prevention. Obstet Gynecol 81(1):131–136, 1993.
5. Gemson DH, Colombotos J, Elinson J, Fordyce EJ, Hynes MM, Stoneburner R: Acquired immunodeficiency syndrome prevention: Knowledge, attitudes and practices of primary care physicians. Arch Intern Med 151:1102–1108, 1991.
6. Abadie R, Hoffman E: Physician practices and attitudes on HIV related issues: A survey of LSMS primary care physicians. J La State Med Soc 144:283–288, 1992.
7. Darr MS, Sinclair AE: HIV disease: Educational needs of Oregon family physicians. Fam Med 24(1):21–23, 1992.
8. Schultz JM, MacDonald KL, Heckert KA, Orterholm MT: The Minnesota AIDS physician survey. Minn Med 71:277–283, 1988.
9. Lofgren SP: Update October 1988: Physician survey about AIDS. J Arkansas Med Soc 85(5):199–202, 1988.
10. Boekeloo BO, Marx ES, Kral AH, Coughlin SC, Bowman M, Rabin DL: Frequency and thoroughness of STD/HIV risk assessment by physicians in a high risk metropolitan area. Am J Public Health 81(12):1645–1648, 1988.
11. Minkoff HL, Landesman SH: The case for routinely offering prenatal testing for human immunodeficiency virus. Am J Obstet Gynecol 159(4):793–796, 1988.
12. Somerville J: Abortion clinics found refusing HIV-infected women. Am Med News 33:4–5, 1990.
13. McIlvaine PJ: In doctor's office, on trial for AIDS. New York Times p 28, September 27, 1987.
14. Nolan K: Human immunodeficiency virus, infection, women, and pregnancy: Ethical issues. Obstet Gynecol Clin North Am 17(3):651–666, 1990.
15. U.S. Department of Health and Human Services: Healthy people 2000. DHHS Publication No. (PHS) 91-502121990. Washington, DC: U.S. Government Printing Office, 1991.
16. ACOG Newsl 37(9):1, September 1993.
